# Identifying strategic priorities for advancing global drowning prevention: a Delphi method

**DOI:** 10.1136/bmjgh-2023-013303

**Published:** 2023-09-14

**Authors:** Justin-Paul Scarr, Jagnoor Jagnoor

**Affiliations:** 1 The George Institute for Global Health, University of New South Wales, Newtown, New South Wales, Australia; 2 Royal Life Saving Society - Australia, Broadway, New South Wales, Australia; 3 Injury Division, The George Institute for Global Health, New Delhi, India

**Keywords:** Health policy, Injury, Qualitative study

## Abstract

**Introduction:**

The burden of drowning is gaining prominence on the global agenda. Two United Nations system resolutions in 3 years reflect rising political support, but priorities remain undefined, and the issue lacks a global strategy. We aimed to identify strategic priorities for advancing global drowning prevention using a modified Delphi method.

**Methods:**

An advisory group was formed, and participants recruited with diverse expertise and backgrounds. We used document review, and data extracted from global health partnerships to identify strategic domains and draft priorities for global drowning prevention. Participants rated the priorities in two Delphi rounds, guided by relevance, feasibility and impact on equity, and where consensus was ≥70% of participants rating the priority as critical.

**Results:**

We recruited 134 participants from research (40.2%), policy (26.9%), technical (25.4%) and community (7.5%) backgrounds, with 38.1% representing low- and middle-income countries. We drafted 75 priorities. Following two Delphi rounds, 50 priorities were selected across the seven domains of research and further contextualisation, best practice guidance, capacity building, engagement with other health and sustainable development agendas, high-level political advocacy, multisectoral action and strengthening inclusive global governance. Participants scored priorities based on relevance (43.2%), feasibility (29.4%) and impact on equity (27.4%).

**Conclusion:**

Our study identifies global priorities for drowning prevention and provides evidence for advocacy of drowning prevention in all pertinent policies, and in all relevant agendas. The priorities can be applied by funders to guide investment, by researchers to frame study questions, by policymakers to contrast views of expert groups and by national coalitions to anchor national drowning prevention plans. We identify agendas including disaster risk reduction, sustainable development, child and adolescent health, and climate resilience, where drowning prevention might offer co-benefits. Finally, our findings offer a strategic blueprint as the field looks to accelerate action, and develop a global strategy for drowning prevention.

**WHAT IS ALREADY KNOWN ON THIS TOPIC**

- Previous studies call for expanded research on drowning burden and solutions across contexts, and for strengthened capacity for multisectoral action.
- The need for framing drowning prevention, in ways to increase prioritisation in non-health sectors, and for inclusive global governance and accountability frameworks, is well established.

**WHAT THIS STUDY ADDS**

- Our study identifies 50 strategic priorities for global drowning prevention, across the domains of: (1) research and contextualisation of drowning prevention, (2) best practice guidance, (3) capacity building, (4) engagement with other health and sustainable development agendas, (5) high-level political advocacy, (6) multisectoral action and (7) inclusive global governance with strengthened accountability.
- Our findings provide a framework to promote multisectoral action for drowning prevention, engage other health and sustainable development agendas and inform the development of a global strategy for drowning prevention.

**HOW THIS STUDY MIGHT AFFECT RESEARCH, PRACTICE OR POLICY**

- Our study provides further evidence for advocacy of drowning prevention in all pertinent policies and agendas, and at global, regional and national levels.
- In policy and practice our research provides a list of issues, ideas and areas in need of attention, and that may have utility across the many stakeholders and disciplines that have potenital to impact on drowning prevention.
- The priorities could be used by funders to guide investment decisions, by researchers to frame study questions in identified gaps, by policymakers to contrast views of expert groups and by national coalitions to anchor national drowning prevention plans. Advocates and agencies working in sustainable development agendas might note our priorities and ask what co-benefits drowning prevention might offer.

## Introduction

Drowning prevention has struggled to gain political prioritisation, despite an estimated 236 000 lives lost annually, 90% in low- and middle-income countries (LMICs), and being among the 10 leading causes of death for people aged 1–24 years, in every region of the world.^1^ Two United Nations system resolutions in 3 years, changes that narrative, and recasts drowning prevention as a legitimate programme in global public health discourse.

The resolutions: United Nations General Assembly (UNGA) Resolution on Global Drowning Prevention, April 2021, A/RES/75/273,^2^ and World Health Assembly (WHA) Resolution, ‘Accelerating action on global drowning prevention’, May 2023 WHA76.18,^3^ raise high-level awareness and emphasise the need for action. The resolutions highlight diverse contexts, which range from increased drowning risk to unsupervised children exposed to water bodies in everyday life, to occupational risk for artisanal fishers, to drowning risk in flood disasters and to drowning in recreational settings in high-income countries. The resolutions reinforce alignment with the Sustainable Development Agenda 2030 and emphasise the need for multisectoral action for drowning prevention.

The UNGA resolution invites WHO, which has increased its drowning prevention efforts since 2014,^4–7^ to support Member States and coordinate actions among UN agencies. In response, the WHA resolution confirmed the establishment of a global alliance for drowning prevention. The global alliance concept is eagerly anticipated, and should focus on unifying multisectoral actors, strengthening cohesion and promoting alignment and accountability, with the most susceptible and affected communities actively engaged in decision-making.^8–11^

This study aims to establish consensus on the strategic priorities for the advancement of drowning prevention over the next 5–10 years, to inform a global strategy for drowning prevention.

## Methods

**Figure 1:**
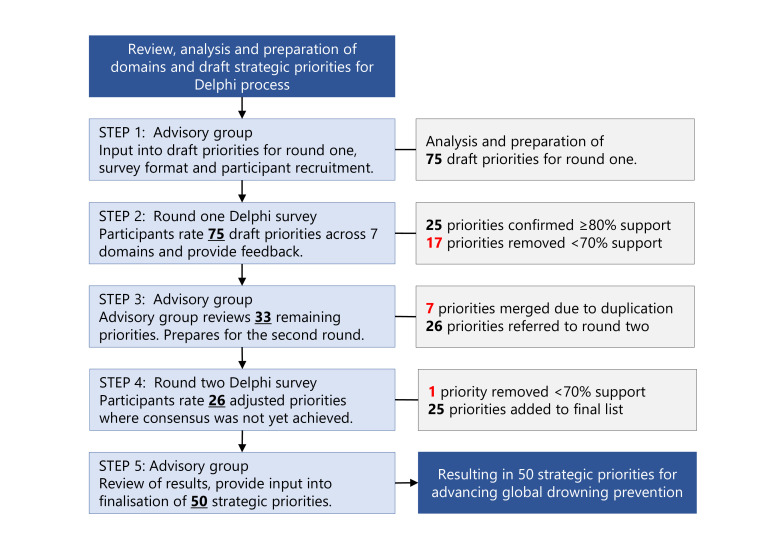
Identifying strategic priorities Delphi method flow chart.

We conducted document review, followed by a five-step online Delphi consensus study (figure 1). The study was guided by an advisory group and informed by the reporting guideline for health research priority setting (REPRISE).^12^

### Document review

Our objectives were twofold: to assess challenges and opportunities in global drowning prevention through comprehensive literature review, and to identify domains and strategic priorities in other partnerships where WHO plays a role to address health and development issues.

First, we searched PubMed, The Cochrane Library, Web of Science, Embase (search terms: ‘drowning’, ‘drowning (MeSH)’ or ‘drowning, near (MeSH)’) and the web databases of WHO, Iris, World Bank, UNICEF (search terms ‘drowning’ or ‘water safety’) to identify areas that contribute to or impede progress of the global agenda for drowning prevention. We included English language articles only but note that future drowning prevention studies need to map scientific and policy content authored in languages other than English, especially as it relates to policy documentation of national governments. The search included reports, empirical studies, policies, strategies and data broadly associated with drowning prevention. We included global and regional level documents published by UN agencies, multilaterals, international non-governmental organisation (NGOs) and development agencies covering multiple countries or regions.

Second, we identified approaches to governance and strategic domains within global partnerships where WHO plays a coordinating or participatory role. We extracted data on priorities, domains and activities from WHO website’s list of partnerships and collaborative arrangements (https://www.who.int/about/collaboration/partnerships). Search results informed the selection of domains and drafting of strategic priorities (online supplemental file 1, 2)

10.1136/bmjgh-2023-013303.supp1 Supplementary data

### Advisory group

We recruited an advisory group (n=6), selecting members from existing research and policy networks of both authors. Advisory group members included technical experts covering research, policy and practice, and a breadth of contexts reflective of study participant groups including from LMICs. Meetings were held virtually.

### Participant recruitment

We used a purposive snowball sampling technique to recruit eligible participants. The eligibility criteria were based on expertise and work context. We included policymakers, researchers, programme managers or advocates for affected communities; and those working in UN agencies, governments, NGOs, foundations, academic institutions or communities affected by drowning; and active within a field with scope for connection to drowning prevention.

Participants were identified through document analysis, existing networks and referrals from participants. The study was promoted by drowning prevention groups who posted an advertisement on social media, in newsletters or by sending an email invitation. We targeted expanded reach to under-represented groups through personal networks, social media postings or email invitations. Participants were directed to the online Participant Information Statement and Consent Form throughout the recruitment phase via a link. Potential participants were required to read the form before providing implied consent by registering for the study and receiving the Delphi survey.

### Data collection and analysis

We used REDCap,^13^ an online software, to develop structured questionnaires for the rating process. Interface testing was conducted to enhance readability and ease of rating. Pilot testing estimated that rounds would take 15–20 mins to complete.

Participants were asked to rate priorities using a 9-point scale; 1–3 being of limited importance, 4–6 being of moderate importance and 7–9 being of critical importance for reducing global drowning burden. Participants had the option to use ‘no-comment’ if unable to rate a priority. Initially, consensus was defined as ≥50% of participants rating a measure as critical (7–9), but it was later adjusted to ≥70% with advisory group agreement, and considering similar study consensus thresholds.^14 15^

At the completion of the first Delphi survey, priorities that reached high levels (≥80%) of consensus were confirmed, and those with lower than 65% support were removed. The remaining priorities were taken into the second-round. In each round, open text fields collected qualitative data.

In round two we asked participants to indicate a guiding principle; relevance, feasibility, equity or all the above, for decision-making in each domain. We described relevance as the extent to which addressing this challenge, issue or solution would reduce the burden of drowning prevention at global, regional or national levels. We described feasibility as the extent to which addressing the challenge, issue or solution is practical or feasible given resources, technical barriers and other support. We described equity as the extent to which addressing the challenge, issue or solution would address drowning prevention in under-resourced populations.

The scores and feedback were tabulated using SPSS and presented for advisory group input. Adjustments between rounds included merging similar priorities, and redrafting based on feedback.

### Patient and public involvement statement

There was no public/patient involvement in this study. Participants did not receive any compensation. They will receive an email link to access the results, unless they opted out of such in the consent process.

### Role of the funding source

The funding source (Australian Government Research Training Program Scholarship) played no role in the design, methods, data collection or analysis of study results.

## Results

### Domains and draft strategic priorities

Following our review, we selected seven domains covering: (1) research and further contextualisation of drowning prevention, (2) best practice guidance, (3) capacity building, (4) engagement with health and sustainable development agendas, (5) high-level political advocacy, (6) multisectoral action (initially split into two sections: (6a) facilitating multisectoral action, and (6b) sectors for multisectoral action) and (7) inclusive global governance with strengthened accountability. We drafted 75 prospective strategic priorities across the domains.

### Characteristics of participants

A total of 134 experts participated from 46 countries. Participant characteristics included community (n=10, 7.5%), policy (n=36, 26.9%), research (n=54, 40.2%) and technical backgrounds (n=34, 25.4%) (table 1). Participation came from LMICs (n=51, 38.1%) and high-income countries (n=83, 61.9%). Participants were from all WHO regions, with the lowest representation from the Eastern Mediterranean region (n=3, 2.2%). The round two response rate was 82.0% (n=110) of round one participants.

**Table 1:** Characteristics of study participants

Respondent’s characteristics	Delphi round 1	Delphi round 2
Number of respondents	134	110
Number of countries represented	46	40
Respondent main areas of relevant expertise (%)		
Community member—lived experience	10 (7.5)	6 (5.5)
Policy or planning	36 (26.9)	29 (26.7)
Research	54 (40.2)	49 (44.5)
Technical service delivery	34 (25.4)	26 (23.6)
Economic category of country status (%)		
High-income country	83 (61.9)	74 (67.3)
Low and middle-income country	51 (38.1)	36 (32.7)
World Health Organization region (%)		
Africa region	18 (13.4)	12 (10.9)
Americas region	26 (19.4)	23 (20.9)
Eastern Mediterranean region	3 (2.2)	3 (2.7)
Europe region	34 (25.4)	29 (26.4)
South-East Asia region	23 (17.2)	17 (15.5)
Western Pacific region	30 (22.4)	26 (23.6)

### Consensus on strategic priorities

In the first Delphi survey we presented 75 strategic priorities across 7 domains of which 25 priorities were rated as critically important by ≥80% of participants (figure 1).

Based on the first-round findings, we excluded 17 priorities that had insufficient support (<70%) and 7 priorities were merged following feedback. Two priorities were scored highly by LMIC participants but failed to meet the round one threshold overall: best practice guidance and capacity for lifeguard (69.5%), and partnerships with the small-scale artisanal fisheries sector (69.6%) and were included in round two based on advisory group advice.

We presented 26 priorities in round two where all but one priority achieved consensus (≥70%) of participants rating them as critical (score 7–9).

We established consensus for 50 strategic priorities, across research and further contextualisation of drowning prevention (n=7), best practice guidance (n=7), capacity building (n=7), engagement with health and sustainable development agendas (n=4), high-level political advocacy (n=5), multisectoral action (n=12) and for inclusive global governance (n=8) (table 2).

**Table 2:** Consensus support and rank of strategic priorities for advancing global drowning prevention

Domain	Priority	% high range		Rank
		Round 1	Round 2	Overall
1. Priorities for research and further contextualisation of drowning prevention. The term ‘investigate’ includes expand research, the synthesis and dissemination of existing research.	Investigate the impact of social and demographic factors on drowning prevention.	83.3	–	16
	Investigate the burden and effectiveness of interventions addressing child and adolescent drowning.	82.5	–	18
	Investigate behavioural factors including risk taking, alcohol and other drug use and use of safety equipment.	75.4	81.9	36
	Investigate drowning prevention contexts in open water environments.	70.9	77.9	43
	Investigate the burden and risk across all life stages, including men and older adults.	73.8	76.0	44
	Investigate the impact of climatic and disaster contexts (including heatwave, flooding, tsunami and storm surge).	77.0	75.5	45
	Investigate global and regional burdens and contexts.	75.4	74.8	47
	Investigate the influence of migration in transit and settlement on drowning and drowning prevention.	62.7	Exc.	
	Investigate the use of physical environmental modification as an intervention for drowning prevention.	55.6	Exc.	
	Investigate occupational contexts including workers employed in small-scale fishing and agriculture settings.	55.1	Exc.	
	Investigate treatment, rehabilitation and psychosocial support for non-fatal drowning.	50.0	Exc.	
2. Priorities for best practice guidance. The term ‘develop’ includes implementation and evaluating.	Develop systematic approaches to teaching children swimming and water safety skills.	90.7	–	1
	Promote best practice guidance for community education and training interventions.	84.7	–	12
	Promote WHO guidance for safe places for preschool children in high burden populations.	83.5	–	13
	Develop best practice guidance for integrating drowning prevention into existing disaster preparation, response and resilience systems.	79.7	83.8	31
	Develop systematic approaches to training community members in safe rescue and resuscitation.	78.0	81.9	35
	Develop best practice guidance and capacity for lifeguard (pool and open water) services, especially in LMICs.	69.5*	81.1	39
	Develop best practice guidance for emergency treatment, hospital care and rehabilitation for drowning victims.	72.4	72.4	49
	Develop best practice guidance for drowning prevention through safe boating, shipping and ferry regulations.	68.6	Inc.	
	Develop best practice guidance for the prevention of alcohol-related drowning in recreational settings.	64.4	Exc.	
3. Priorities for capacity building. The phrase ‘strengthen capacity’ includes developing, resourcing and evaluating.	Strengthen capacity for data systems to measure and monitor drowning burden at all levels.	88.8	–	2
	Strengthen capacity for policy-based approaches to drowning prevention.	80.0	–	25
	Strengthen capacity for implementation research.	79.1	84.5	29
	Strengthen capacity for behaviour change and social marketing.	73.3	84.3	30
	Strengthen capacity of safety and risk management systems.	71.1	83.2	33
	Strengthen capacity of civil society to engage in drowning prevention (at all levels).	75.2	79.8	40
	Strengthen capacity for multidisciplinary research.	73.9	73.8	48
	Strengthen the capacity of the health sector for drowning prevention.	68.7	Inc.	
	Strengthen technical capacity for lifeguard and rescue services (pool and open water).	68.4	Inc.	
	Strengthen technical capacity of media sector to investigate and report on drowning prevention.	63.8	Exc.	
4. Priorities for engagement with health and sustainable development agendas.	Align drowning prevention within the Disaster Risk Reduction agenda and to the Sendai Framework.	85.1	–	9
	Align drowning prevention with the Sustainable Development Agenda 2030.	82.3	–	20
	Integrate drowning prevention within child and adolescent health agendas.	77.7	92.3	26
	Integrate drowning prevention within climate and health agendas.	74.6	83.5	32
	Integrate drowning prevention within Disaster Risk Reduction agendas.	81.6	Inc.	
	Integrate drowning prevention within SDG 3 - good health and well-being agendas.	67.3	Exc.	
	Integrate drowning prevention within Universal Health Coverage agendas.	61.5	Exc.	
	Integrate drowning prevention within the United Nations Decade of Healthy Ageing (2021–2030).	55.1	Exc.	
	Integrate drowning prevention within planetary health agendas.	52.8	Exc.	
	Integrate drowning prevention within urban health agendas.	51.8	Exc.	
5. Priorities for high level political advocacy.	Develop strategies for engagement with the Least Developed Countries, Landlocked Developing Countries and Small Island Developing States.	82.9	–	17
	Develop an accountability framework aligned to the UN Resolution on Global Drowning Prevention to monitor national progress.	80.7	–	24
	Develop strategies for engagement including with UN agencies like UNICEF, UNISDR, IMO, ILO, IOM, UNDP.	76.8	86.9	27
	Develop strategies for continued engagement at the World Health Assembly.	77.5	86.1	28
	Develop strategies for engagement with World Bank initiatives for urban, rural, environmental and human development.	73.2	78.0	42
	Develop strategies for engagement with the UN Office for Disaster Risk Reduction, and alignment to the Sendai Framework.	76.9	Inc.	
	Develop strategies to transform the UN Resolution voluntary actions into an accountability, reporting and measurement framework.	70.1	Inc.	
	Develop strategies to position drowning prevention within the next report of the Intergovernmental Panel on Climate Change.	67.0	Exc.	
	Develop strategies for integrating drowning prevention into other UN plans or strategies, please specify in notes.	66.3	Exc.	
	Develop strategies for engagement with the International Olympic Committee Peace and Development through Sport Initiative	43.1	Exc.	
6. Priorities for multisectoral action for drowning prevention.	Develop national/subnational strategies and action plans.	87.7	–	4
	Develop inclusive approaches with governments, civil society, community and private sector.	86.5	–	6
	Strengthen multisectoral policies that address drowning prevention challenges (at all levels).	85.8	–	7
	Strengthen coordinating mechanisms to facilitate collaboration and accountability at national/subnational levels.	85.0	–	10
	Prioritise partnerships with the early childhood care and development sector.	83.5	–	14
	Prioritise partnerships with the education sector.	82.5	–	19
	Prioritise partnerships with the emergency sector, including with police and fire departments.	81.6	–	22
	Increase funding for pathway projects in multisectoral action for drowning prevention.	82.1	–	21
	Prioritise partnerships with the health and well-being sector.	72.2	81.6	38
	Prioritise partnerships with the maritime, boating and passenger ferry sector.	79.1	78.6	41
	Prioritise partnerships with the transport sector, including water transport and roads.	72.8	75.0	46
	Prioritise partnerships with the small-scale artisanal fisheries sector.	69.6*	71.6	50
	Prioritise partnerships with the tourism sector.	68.7	Exc.	
	Prioritise partnerships with the sport, physical activity and leisure sector.	63.5	Exc.	
	Prioritise partnerships with the water, sanitation and hygiene sector.	63.1	Exc.	
	Prioritise partnerships with the agriculture and rural development sector.	62.6	Exc.	
7. Priorities for inclusive global governance with strengthened accountability.	Increase funding for advocacy, research, policy and implementation.	88.7	–	3
	Develop regional strategies, and action plans for drowning prevention.	86.8	–	5
	Establish a global alliance for drowning prevention.	85.8	–	8
	Prioritise inclusion of high burden populations in decision-making and global governance.	85.0	–	11
	Convene multistakeholder meetings and forums to coordinate drowning prevention efforts.	83.5	–	15
	Adopt a targeted country approach, by selecting 10 priority countries across different contexts to identify and share novel approaches.	81.4	–	23
	Develop a global strategy for drowning prevention.	73.7	82.5	34
	Develop a business case for establishing a global fund for drowning prevention.	73.5	81.7	37

Note: * priority included in round two as advisory recommendation as scored highly by LMIC participants in round one.

Exc, excluded after round one; ILO, International Labour Organization; IMO, International Maritime Organization; Inc, included in another priority after round one; IOM, International Organization for Migration; LMICs, low and middle-income countries; MSA, multisectoral action; SDG, Sustainable Development Goal; UN, United Nations; UNDP, United Nations Development Programme; UNISDR, United Nations Office for Disaster Risk Reduction.

#### Research and further contextualisation of drowning prevention

In round one, we presented 12 draft priorities in the research and further contextualisation of drowning prevention domain, with focus on challenges across populations, environments and activities.

In round one, research investigating social and demographic factors and both the burden and the effectiveness of interventions addressing child and adolescent drowning were rated highly. In round two, research investigating behavioural factors including risk taking, alcohol and other drug use, and use of safety equipment were rated highly.

Four research priorities did not meet the criterion in round one and were removed. The most notable being investigating the influence of migration in transit and settlement on drowning prevention, where low levels of support, especially among researchers and technical experts, meant that the priority was removed.

#### Best practice guidance

We identified nine draft priorities in the best practice guidance domain, which we described as techniques, methods or approaches that through research or experience have proven to support drowning prevention.

In round one, the highest rated priorities were systematic approaches to teaching children swimming and water safety skills, and community education and training interventions. In round two, guidance for integrating drowning prevention into existing disaster preparation, response and resilience systems was rated highly. We removed the priorities for guidance for safe boating, shipping and ferry regulations, and alcohol-related drowning in recreational settings in round one as both were below the inclusion criterion.

#### Capacity building

We identified 10 draft priorities for capacity building, which we describe as the process of developing and strengthening skills, processes, and resources that organisations and communities need to avoid, reduce, mitigate against drowning.^16^

In round one, the strengthening of capacity for data systems to measure, map and monitor drowning burden at all levels was the highest rated priority, followed by capacity for policy-based approaches to prevention, and capacity for implementation research in round two. The priority for strengthening of capacity for lifeguard and rescue services (pool and open water) received low level of support in round one and was omitted.

#### Engagement with health and sustainable development agendas

We identified nine draft priorities in the engagement with health and sustainable development agendas domain.

In round one, engagement with the Disaster Risk Reduction agenda and to the Sendai Framework, and engagement with the Sustainable Development Agenda 2030,^17^ scored highly. Four agendas; Universal Health Coverage, the UN Decade of Healthy Ageing, planetary and urban health agendas were removed in round one.

In round two, child and adolescent health, and climate and health agendas reached the inclusion criterion.

#### High-level political advocacy

We identified 10 draft priorities in the high-level political advocacy domain, which is considered important to accelerating and sustaining progress.

In round one, strategies for high-level advocacy of drowning prevention for the least developed countries, landlocked developing countries and small island developing states and the development of an accountability framework aligned to the UN Resolution on Global Drowning Prevention, achieved strong consensus. The draft priority seeking to implement strategies for engagement with the International Olympic Committee Peace and Development through Sport Initiative scored the lowest of any priority and was removed in round one.

In round two, engagement with UN agencies like UNICEF, United Nations office for Disaster Risk Reduction International Maritime Organization (IMO), International Labour Organization, International Office for Migration and United Nations Development Programme (UNDP) rated highly.

#### Multisectoral action for drowning prevention

The multisectoral action for drowning prevention domain was initially presented in two subdomains: priorities for facilitation of multisectoral action and sectors for multisectoral action.

We identified five draft priorities in the subdomain for facilitation of multisectoral action, aiming to address the notion that successful multisectoral action needs deliberate activities, specific techniques and governance aimed at leveraging the strengths of diverse sectors for collective impact.^18 19^ The development of national/subnational-level strategies, and approaches that include governments, civil society, community and private sector in multisectoral action for drowning prevention rated highly.

We identified 12 draft priorities in the subdomain of sectors for multisectoral action. In round one, partnerships with the early childcare and development, education and the emergency response sectors, including with police and fire departments, achieved strong consensus. Four sectors were removed after round one including 1) tourism, 2) sport, physical activity and leisure, 3) water, sanitation and hygiene, and 4) agriculture and rural development.

#### Inclusive global governance

We identified eight draft priorities for inclusive global governance with strengthened accountability. All priorities achieved greater than 80% consensus in round one or two and were confirmed.

In round one, participants prioritised increased funding, development of regional strategies, establishing a global partnership or alliance and the inclusion of groups from high burden populations in decision-making and global governance. Developing a global strategy for drowning prevention, and a business case for establishing a global fund for drowning prevention were added after round two.

### Priorities ranked by context and expertise

The priorities were analysed and ranked across all participants groups (table 3). The highest priorities were (1) develop systematic approaches to teaching children swimming and water safety skills, (2) strengthen capacity for data systems to measure, map and monitor drowning burden at all levels, (3) increase funding availability for advocacy, research, policy and implementation, (4) develop national/subnational-level strategies, and (5) develop regional strategies, and action plans for drowning prevention.

**Table 3:** Top 25 ranked priorities by setting and expertise

Domain	Priority	Context			Expertise			
		Overall	HIC	LMIC	Technical	Policy	Research	Community
Best practice guidance	Develop systematic approaches to teaching children swimming and water safety skills.	1	2	3	3	8	1	1
Capacity building	Strengthen capacity for data systems to measure and monitor drowning burden at all levels.	2	4	1	4	16	2	6
Inclusive global governance	Increase funding for advocacy, research, policy and implementation.	3	5	2	6	9	3	7
Multisectoral action	Develop national/subnational strategies and action plans.	4	1	19	14	1	6	24
Inclusive global governance	Develop regional strategies, and action plans for drowning prevention.	5	9	4	7	10	10	10
Multisectoral action	Develop inclusive approaches with governments, civil society, community and private sector.	6	8	6	15	14	4	17
Multisectoral action	Strengthen multisectoral policies that address drowning prevention challenges (at all levels).	7	12	7	16	3	14	19
Inclusive global governance	Establish a global alliance for drowning prevention.	8	7	11	1	13	18	13
Engagement with health and sustainable development agendas	Prioritise partnerships in the Disaster Risk Reduction sector aligned to the Sendai Framework.	9	10	12	12	2	20	15
Multisectoral action	Strengthen coordinating mechanisms to facilitate collaboration and accountability at national/subnational levels.	10	3	20	20	5	8	18
Inclusive global governance	Prioritise inclusion of high burden populations in decision-making and global governance.	11	13	13	17	11	13	11
Best practice guidance	Promote best practice guidance for community education and training interventions.	12	15	9	11	18	16	3
Best practice guidance	Promote WHO guidance for safe places for preschool children in high burden populations.	13	19	10	18	24	7	2
Multisectoral action	Prioritise partnerships with the early childhood care and development sector.	14	6	22	24	12	9	9
Inclusive global governance with strengthened accountability	Convene multistakeholder meetings and forums to coordinate drowning prevention efforts.	15	23	5	8	17	19	14
Research and further contextualisation of drowning prevention	Investigate the impact of social and demographic factors on drowning prevention.	16	18	15	10	20	17	5
High-level political advocacy	Develop strategies for engagement with the Least Developed Countries, Landlocked Developing Countries and Small Island Developing States.	17	22	8	19	25	5	8
Research and further contextualisation of drowning prevention	Investigate the burden and effectiveness of interventions addressing child and adolescent drowning.	18	16	21	25	15	12	4
Multisectoral action	Prioritise partnerships with the education sector.	19	11	23	9	4	24	22
Engagement with health and sustainable development agendas	Align drowning prevention with the Sustainable Development Agenda 2030.	20	17	18	5	7	25	23
Multisectoral action	Increase funding for pathway projects in multisectoral action for drowning prevention.	21	14	24	21	19	11	25
Multisectoral action	Prioritise partnerships with the emergency sector, including with police and fire departments.	22	21	16	2	21	22	21
Inclusive global governance	Adopt a targeted country approach, by selecting 10 priority countries across different contexts to identify, and share novel approaches.	23	25	14	22	23	15	12
High-level political advocacy	Develop an accountability framework aligned to the UN Resolution on Global Drowning Prevention to monitor national progress.	24	24	17	13	22	23	16
Capacity building	Strengthen capacity for policy-based approaches to drowning prevention.	25	20	25	23	6	21	20

HIC, high-income country; LMIC, low- and middle-income country; UN, United Nations.

The priorities were analysed by setting and expertise (table 3). LMIC participants ranked the need to develop national/subnational level multisectoral drowning prevention and water safety plans (rank 4) more highly than high-income country (HIC) participants (rank 19). Research experts ranked the strengthening of capacity for data systems to measure, map and monitor drowning burden at all levels as of critical importance (rank 2), whereas policy experts ranked the priority lower (rank 16). Prioritising partnerships in disaster risk reduction aligned to the Sendai Framework, was rated much more highly by policy experts (rank 2) than any other constituent. Technical experts ranked the need to establish a global alliance for drowning prevention as their highest priority (rank 1).

### Guiding factors

In round two, participants were asked to indicate the guiding factor used to determine their scores in each domain. Overall participants scored priorities based on relevance (43.2%), feasibility (29.4%) and impact on equity (27.4%). There was minor variation across domains, except the high-level political advocacy domain where each factor was applied more evenly (online supplemental file 3)

## Discussion

To our knowledge this is the first multistakeholder and geographically diverse consultation on strategic priorities for drowning prevention, and it comes at a time where increased high-level political focus brings new energy, resources and diverse interests to this previously underappreciated health and development challenge. Recent studies,^1 8 9 20^ as well as global reports by WHO, have called for multisectoral, and multistakeholder approaches. These calls are reinforced in successive UN,^2^ and WHA,^3^ Resolutions, the most recent resulting in WHA Member States requesting that WHO establish a global alliance for drowning prevention, underpinned by a new sense of purpose, strategy and cohesion—globally, regionally and in some cases nationally. It is in this context that we aimed to identify strategic priorities for advancing drowning prevention, by engaging diverse experts from across research, policy and practice.

### Multisectoral action beyond health

Recognition of the need for multisectoral action for drowning prevention is growing.^9 20^ Three of the top 10 strategic priorities focus on facilitating multisectoral action, and the highest levels of support were from policy experts. Priorities include investing in national and subnational coordination, the development of accountability frameworks, using whole-of-society approaches inclusive of governments, civil society, community members and the private sector, and increasing focus on drowning prevention in all policies. The importance of policy intersections with and between non-health sectors has been reinforced previously. Our study points to the strategic importance of engaging non-health sectors including disaster risk reduction, child development, education and emergency response in drowning prevention.

Policy coordination is likely to be a key challenge in advancing drowning prevention. Where cross-sectoral coordination is currently assigned by national governments, the ministries chosen are not homogenous across countries or regions. For example, drowning prevention is led by the Ministry of Labour, Invalids and Social Affairs in Vietnam, Ministry of Public Health in Thailand and Department of Fire and Rescue in Malaysia. Should a future UNGA convene a ministerial conference on drowning prevention, it is highly likely that the ministers represented could span the full spectrum of government sectors. This heterogeneity adds intrigue to decisions around core skill sets for capacity building and makes the sharing of lessons across countries both critical and complex. The richness and potential for interactions across sectors may function as an important social experiment and should be investigated further to strengthen drowning prevention action and inform other multisectoral policy agendas. There is scope for drowning prevention researchers and policymakers to investigate how other issues deal with complexities when housed within different government departments, for example, road traffic injury can be transport and/or public health, including through use of public–private partnerships.

### Positioning drowning in other health and development agendas

Navigating drowning prevention in the context of the Sustainable Development Goals (SDGs), and other key global development agendas presents challenges and requires a clear set of objectives and a plan of action. Consensus identified four development agendas where integration may impact on drowning prevention.

First, pursuing partnerships in disaster risk reduction with alignment with the Sendai Framework,^21^ represents the most significant opportunity. Flooding is a major contributor to drowning risk and studies reinforce the need for preparedness for water-related disasters,^22^ flood rescue training for emergency personnel,^23^ and risk analysis and early warning systems. Sendai promotes subnational and local government disaster risk reduction planning, where drowning prevention should form part of multihazard approaches to building local disaster resilience. Flood and coastal inundation risk mapping should be expanded to chart other known drowning hazards. Understanding barriers and expanding investments in locally generated disaster risk data could contribute to and strengthen surveillance for drowning prevention.^24 25^ National and community level disaster coordination could be adapted to include mechanisms with potential to extend impacts on drowning prevention outside of disaster events.

Second, alignment to the Sustainable Development Agenda 2030 and the SDGs is strongly supported and is known to have had positive influence on policy support during the UNGA resolution process.^8^ The implementation of pathfinding research and programmes across identified SDGs is much needed and may form a strong basis for further multisectoral action for drowning prevention. Previous analysis identifies the need for drowning prevention to look outside of its silos and engage across the SDG framework.^26^ Positioning drowning within other SDG agendas, requires action across domains including capacity building, and research contextualising drowning prevention for integration within targeted agendas.

Drowning prevention advocates should not lose sight of the fact that the SDGs are nearing an end (2030), and the process to develop a subsequent agenda will commence in coming years. Strategies to enhance the positioning of drowning prevention in future sustainable development strategies requires planning and investment now.

Third, participants prioritised partnering with child and adolescent health agendas, where the known impacts on children, and successful interventions like daycare and survival swimming present continued opportunities.^27 28^ Although the relative mortality burden is much higher in children 1–4 years, there is recognition of the need to address adolescent cohorts.^29^ The key to addressing adolescent drowning could be with partners from outside of the health sector, potentially through sport, and agendas that support youth culture, as well as including youth in decision-making, especially in LMICs.^30^ Addressing social risk and protective factors might have utility in adolescent drowning contexts.^31^

Fourth, several domains acknowledged the significance of integrating drowning prevention within climate and health agendas. Research on drowning prevention in the intersections between climate and disaster, and for social and economic factors in populations with a high burden of drowning is essential.^32^ Studies should assess overlapping hazards, exposures and vulnerabilities which may translate to increased drowning risk, whether in household or work settings. Drowning risk in artisanal fisheries,^33 34^ is one such example, where initiatives targeting climate, disaster and enhanced economic outcomes, might also have co-benefits for drowning prevention. Other agendas may offer modalities providing cost-effective and accelerated access to communities with high drowning burden.

### Building inclusive governance

Our study reinforces the importance of building inclusive global governance and strengthened accountability. The emergence of drowning on global health agendas, has been attributed to a small and cohesive coalition of actors, who advocated for drowning prevention in the absence of any formal governance.^8^

Our study identified high levels of participant support for the formation of a global level alliance for drowning prevention. In July 2023, WHO announced the establishment of a Global Alliance for Drowning Prevention. The initial membership consists of five UN agencies; IMO, the UNDP, the Food and Agriculture Organization of the United Nations (FAO), UNICEF and WHO, and five non-state actors: the Royal Life Saving Society - Australia; Royal National Lifeboat Institution (RNLI), UK; the Centre for Injury Prevention and Research, Bangladesh; the George Institute for Global Health; and Bloomberg Philanthropies, who together must take on the responsibility of building inclusive models for governance.

Governance priorities also called for increased funding for advocacy, research, policy and implementation, the inclusion of groups from high burden populations in decision-making and the development of contextually relevant regional action plans.^4 5^ Regional planning often faces resistance within the health field due to several challenges, including governance structures, competing interests and underlying discrepancies between the needs of global and national agendas.^35 36^ To counter this, a global strategy should emphasise contextual differences across and within regions, drive regional collaboration but aim for national level momentum.

Drowning prevention requires deliberate actions to create multistakeholder platforms that include multilaterals, governments, policy, civil society, academic and donor groups in decision-making, meaning the importance of the global alliance cannot be understated. A challenge rests in how, and whether combining diverse sector perspectives at global level, may influence the nature and momentum for community level collaboration. Opportunities may exist where global alliance members have resources, networks or programmes already situated in high drowning burden communities and countries. For example, where FAO is working on climate resilience in an artisanal fishery community where a drowning burden can be identified—can drowning prevention interventions be successfully embedded to protect children from drowning in water near households?

### Informing global strategy

**Figure 2:**
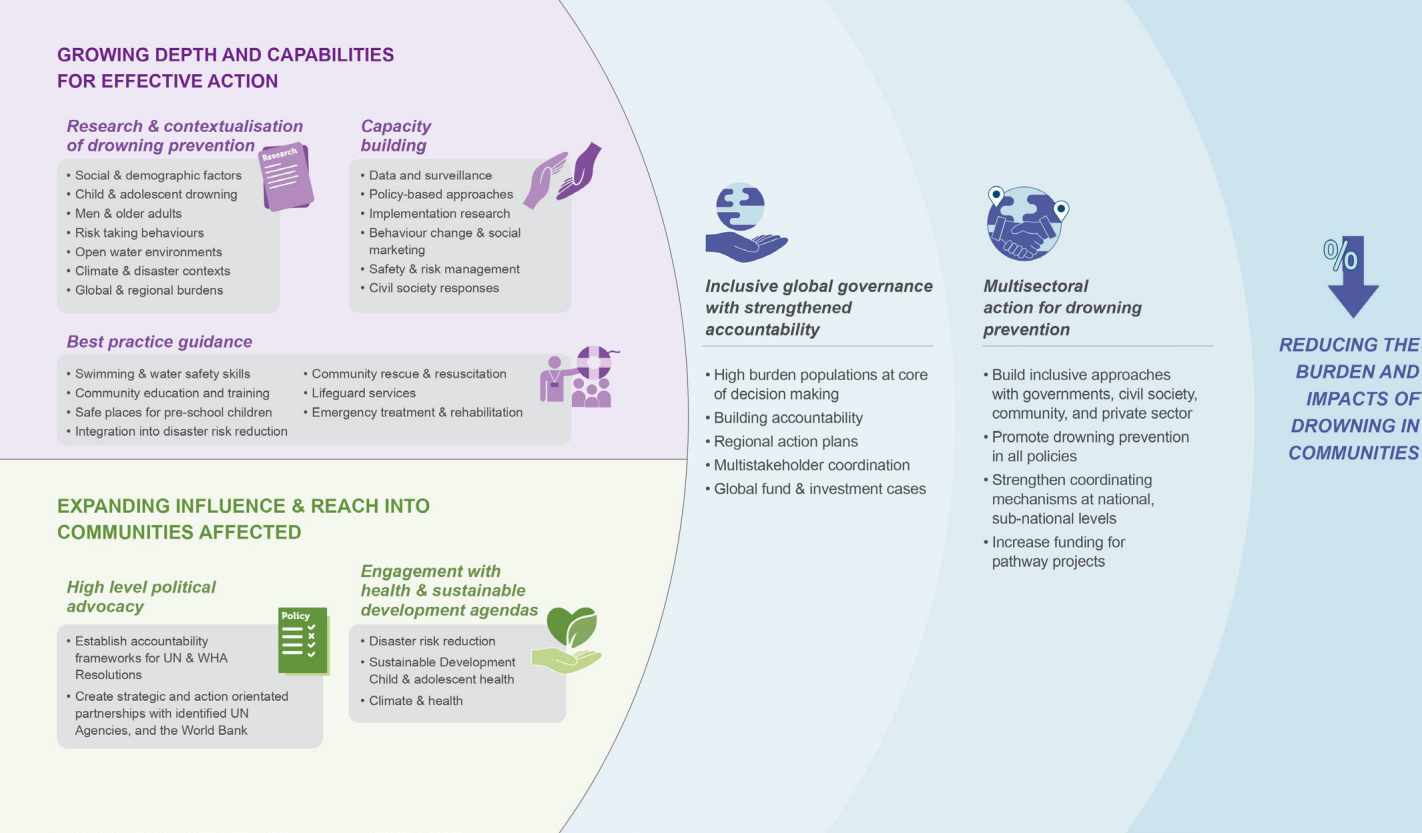
Towards a global strategy for drowning prevention.

Other health and sustainable development agendas have advanced their objectives through multistakeholder partnerships, complimented by a global strategy. WHO indicated that the development of a global strategy for drowning prevention is among the initial tasks of the global alliance. A global strategy for drowning prevention could include each of the domains in this study, and further prioritisation and selection from the strategic priorities identified (figure 2). However, the strategy development process itself offers important benefits that could contribute to actor cohesion, strengthening of partnerships and provide the potential to engage communities at higher risk of drowning in priority setting. High-level progress should be measured by resource generation and mobilisation, and subsequent impacts on drowning prevention at community level.

### Research agenda

Growth in drowning prevention research has been rapid, and its role in understanding issue characteristics critical to generating political awareness.^8^ LMIC participants ranked strengthening capacity for data systems to measure, map and monitor drowning burden at all levels as the highest priority. Investments in drowning data surveillance, using verbal autopsy, has contributed insights in Bangladesh,^37^ India,^38 39^ and Vietnam,^40^ and the methodology has extended to Ghana, Tanzania,^41^ and Uganda,^42^ where studies show drowning rates higher than previously reported.

Data collection in the WHO Western Pacific and South East Asia regional status reports,^4 5^ highlights the depth of the national surveillance challenge. Pockets of drowning data are commonly held across multiple ministries; disaster management may carry flood related deaths, maritime—ferry incidents, police—death certificates and health—data from health facilities. Non-fatal drowning data may not be discernible from other data held by social protection units, and many more cases go unreported. The fact that drowning data exists should bring hope and again reinforces the importance of multisectoral approaches, but it does come with equal parts of risk and opportunity. The objective of developing a perfect national registry for drowning, should be careful not to stamp out enthusiasm, or interest in data collection by non-health actors. Data collection by these sectors may provide sector specific metrics and underline important roles in prevention. For example, coastal boating incidents serve as an important indicator for maritime safety and inform resource allocation and policy implementation, thereby contributing to overall efforts for drowning prevention.

Beyond national estimates, the priority for research investigating social and demographic factors is consistent with calls to focus on the social determinants of drowning and to look upstream for drowning prevention measures, particularly for children and adolescents.^43–45^ Many of the case studies, and evidence based interventions instrumental in elevating drowning in global discourse can be traced to research investments in south-east Asia, and western pacific regions.^8 46^ The search for context in other regions has the potential to generate new insights into drowning prevention, and result in further demands for intervention evidence.

The contrasting perspectives between research, policy and technical experts need further consideration. Significant variances included that the need for research across life stages and in open waters environments including beaches, rivers and lakes, were rated much lower by research experts than policy or technical experts. Several studies point to a much greater need for intervention research compared with further epidemiological studies, especially in well studied areas in HIC settings.^47 48^ In any case, further development of a research agenda for drowning prevention is critical, and must be developed in consultation and based on the needs of policymakers and practitioner communities. Extending involvement in research agenda development to those in sectors yet to be engaged in drowning prevention, may yield further benefits.

### Discord on migrant and refugee drowning

The low rating, and ultimate exclusion, of actions for drowning prevention for migrants in transit and settlement may reflect an uncertain burden, weaknesses in existing approaches,^49^ and a discomfort with political determinants.^8^ This priority was scored slightly higher by LMIC participants than HIC participants but that was not enough for it to be included. It also reached the threshold for inclusion based on community and policy participants but was scored poorly by research and technical participants. Based on community and policy expert interest, the known drowning burden and the urgent need for action as identified in UN reports,^50 51^ the drowning prevention field cannot afford to ignore this critical issue and further policy work in this area is a necessity.

### Variance on priorities among experts

Several domains showed significant difference in viewpoints among experts, sometimes enough for a priority to be excluded in our study results. The requirement for guidance for alcohol-related drowning in recreational settings was rated as important by technical experts, but less important by research and policy experts, so was not included. Alcohol is a significant factor in adult drowning in Australia,^52 53^ and Europe,^54–56^ occupational related drowning and injury in the fishing industry,^57 58^ and is an unknown yet speculated factor in some LMICs.

The differences between LMIC and HIC participant priorities were most prominent in the areas of multisectoral action and strengthened governance, where LMIC participants were more likely to support convening multistakeholder meetings and forums and were less supportive of the need for national and subnational drowning prevention plans than HIC participants. The need for funding and resources was also more important to LMIC participants.

Overall, differences across expert groupings for priorities provides insight into roles and relationships across sectors and expertise. This variation reinforces the need for multidisciplinary approaches and geographically diverse participation in global priority setting, and community engagement when priorities are intended to affect change at local levels. Our study reinforces the value of Delphi method in priority setting activities, specifically as a tool combining the feedback from large heterogenous stakeholder groups.

### Further refinement of global, national and community priorities

Our study has resulted in a longer list of priorities than expected, and in some respects the length of our list undermines the notion of strategic priorities. We take the view that several factors make a comprehensive list vital for the advancement of drowning prevention at this point in its development. First, the multidisciplinary and multisectoral nature of drowning prevention makes an inclusive list important to ensuring engagement and the support of such multifarious interests. Second, our priorities are framed at a global level and need to allow for emerging recognition of contextual diversity in both drowning burden and opportunities for prevention at regional, national and community levels. The emergence of drowning prevention over the past two decades has largely been driven by knowledge generated from a limited number of countries in the Asian continent,^8 46^ the next may be driven by advances in Africa and/or small island nations where burdens are thought to be high, and contexts different. Finally, both the domains and the priorities have potential to be further refined and prioritised at national, subnational and community level. This latter point, ensuring that global priorities result in community impacts, is something that should be built into a global strategy for drowning prevention.

### Limitations

Delphi method has been implemented in previous drowning prevention studies,^59 60^ and for establishing priorities for interpersonal violence prevention,^61^ global eye health,^62^ and harmful gambling.^63^ However, Delphi method is not without weaknesses. Potential weaknesses in panel recruitment were addressed by considering five aspects: panel size, expertise level, heterogeneity, interest and access. Our 134-participant panel achieved balance in expertise and interests but would have been strengthened with increased participants from the Eastern Mediterranean region and from governments. The planned WHO global status report for drowning prevention (expected end 2024), which principally engages governments may increase awareness and engagement in underrepresented nations.

Delphi method has been criticised for potential impacts of bias, which we attempted to minimise by increasing the diversity of participants, achieving a large panel size, and providing and analysing open text fields. Every effort was made to ensure representation from LMIC groups, but the proportion is less than the drowning burden for these areas, and is perhaps a reflection of gaps in existing capacity for drowning prevention.^64^ The way in which we framed the priorities, including that the terminology varied across domains, may have influenced the scoring by participants.

Our consensus definition established a priori, was ≥50% of participants rating a priority of high range importance. This was adjusted to ≥70% after round one in consultation with the advisory group as it was deemed to be too low. A review of 98 Delphi studies found consensus threshold was often set arbitrarily and not reported, and when it was reported a median consensus threshold was ≥75%.^14^

Our study was conducted in English, so may have excluded non-native English speakers. We surveyed preferences for languages other than English and while noting that each completed the survey, 24.6% suggested they preferred a language other than English. The highest was Bangla, (4.6%) followed by French (3.7%), a Chinese language (3.0%), Portuguese (3.0%) or Spanish (3.0%).

## Conclusion

Our study identifies strategic priorities for global drowning prevention and provides evidence for advocacy of drowning prevention in all pertinent policies, and in all relevant agendas. While framed as global priorities, our research provides a list of issues, ideas and areas in need of attention, and that may have utility across the stakeholders and disciplines that contribute to drowning prevention at regional, national and community levels. Applications of the findings of our study include: (1) national coalitions can use the priority domains to anchor national drowning prevention policy and planning, (2) the priorities could be used by funders to guide investment policies and decisions, (3) researchers might frame study questions in identified gaps, (4) policymakers might find insight in contrasting views of the expert groups who participated and (5) advocates and organisations working in the health and sustainable development agendas named might note our priorities and ask what value drowning prevention might offer to their objectives, whether for disaster risk reduction, sustainable development, child and adolescent health or climate resilience. Finally, our findings offer a blueprint to advance global drowning prevention as the field looks to accelerate action, establish a global alliance and develop a global strategy for drowning prevention.

## Data Availability

All data relevant to the study are included in the article or uploaded as supplementary information.
