# Supplementary material for: Identifying strategic priorities for advancing global drowning prevention: a Delphi method
Source: BMJ Glob Health. 2023 Sep 14;8(9):e013303. doi: 10.1136/bmjgh-2023-013303 (PMC10503336; doi:10.1136/bmjgh-2023-013303)
Supplement: Supplementary data [file bmjgh-2023-013303supp001.pdf]

SUPPLEMENT

Identifying strategic priorities for advancing global drowning prevention: a Delphi-method

Authors

Mr. Justin-Paul Scarr, MBA; Royal Life Saving Society—Australia, Sydney, Australia and The George Institute for Global Health, University of New South Wales, Sydney, Australia.

Dr Jagnoor Jagnoor, PhD; The George Institute for Global Health, New Delhi, India and The George Institute for Global Health, University of New South Wales, Sydney, Australia.

Corresponding Author

Mr. Justin-Paul Scarr

Royal Life Saving Society—Australia, PO Box 558, Broadway, NSW, Australia

The George Institute for Global Health, University of New South Wales, Level 5, 1 King Street, Newtown 2042 NSW, Australia

Email : jscarr@georgeinstitute.org.au

Phone : +61 408 434 011

Supplement 1: Selected global heath partnerships where WHO plays a role

Name	Est.	Area of Focus	Decision Making Body	Stakeholders	WHO Role
BreatheLife	2016	Global campaign against air pollution	Committee	WHO, UNEP, CCAC, World Bank	Joint initiative
Climate and Clean Air Coalition	2012	Partnership to reduce short-lived climate pollutants	High Level Assembly	Governments, intergovernmental organizations, businesses, scientific institutions, and civil society	Member
End Violence Against Children	2016	Partnership and Fund to end violence against children	Board of directors	Governments, UN agencies, research institutions, international NGOs, foundations, local CSOs, private sector groups and faith networks	WHO on Board and Exec Committ ee
Every Women Every Child	2010	Movement to address the major global health challenges facing women, children and adolescents	High Level Panel	Governments, multi-laterals, the private sector and civil society	Technical Partner
Global Network for Health in All Policies	2017	Network to strengthen the Health in All Policies (HiAP) approach	Executive Committee	Governments, academia, civil society, United Nations agencies	Member
Global Road Safety Partnership	1999	Not for profit partnership for global road safety	Steering Committee	multi- and bi-lateral development agencies, governments, businesses and civil society organizations.	Member
H6 Partnership	2016	UN partnership to advance the Every Woman Every Child (EWEC) Strategy	Simple affiliation	UNFPA, UNICEF, UN Women, WHO, UNAIDS, World Bank	Key Member
Roll Back Malaria	1998	Platform for coordinated action against malaria	Board of directors	500 partners, including malaria endemic countries, their bilateral and multilateral development partners, the private sector, nongovernmental and community-based organizations, foundations, research, and academic institutions	Mandate d board position
The Global Fund	2002	Partnership to fund efforts the end of AIDS, tuberculosis, and malaria as epidemics	Board of directors	governments, civil society, technical agencies, the private sector and people affected	Technical Partner
Violence Prevention Alliance (VPA)	2004	Network working to prevent violence	Steering Committee	WHO Member State governments and other institutions, including non-governmental and community-based organizations, and private, international, and inter-governmental organizations	Secretari ate
UHC2030	2016	Movement to build health systems for Universal Health Coverage (UHC)	Steering Committee	countries, civil society organisations, foundations, private sector, multilaterals, co-host orgs inc. WHO, World Bank, OECD	Co-host
UN Road Safety Collaboration	2004	Informal mechanism to facilitate cooperation on road safety	Steering Committee	Members States, UN agencies, Development Banks, and other global road safety stakeholders from NGOs, academia, philanthropic foundations, and private sector	Convenor , secretaria te
UN Water	2003	Partnership to coordinate UN entities and international organizations working on water and sanitation issues	Joint Steering Group	UN agencies, programmes and funds with a water-related mandate are Members of UN-Water. Partners are international organizations, professional unions, associations, or other civil-society groups	Member
Working for Health	2016	Joint programme to expand and transform the health and social workforce	Steering Committee	International Labour Organization (ILO), Organisation for Economic Co-operation and Development (OECD), and the World Health Organization (WHO)	Secretari ate
Stop TB Partnership	2001	UN Hosted partnership to revolutionize the TB space and end TB by 2030	Board of directors	Governmental organizations, Donors and partners, NGOs, Private sector, People affected by TB (CSOs, patient organizations, etc), Academia (public sector, etc.)	Secretari ate

Supplement 2: Global strategies identified in review partnerships where WHO is known to play a key role

Title	Date	High Level Statement	Published
WHO global strategy on health, environment, and climate change: the transformation needed to improve lives and wellbeing sustainably through healthy environments	2019	The vision of this WHO global strategy is a world in which sustainable development has eliminated the almost one quarter of the disease burden caused by unhealthy environments, through health protection and promotion, good public health standards, preventive action in relevant sectors and healthy life choices, and which manages environmental risks to health. Key sectors fully integrate health into their decision-making process and maximize societal welfare.	WHA
Climate and Clean Air Coalition 2030 Strategy	2020	Our vision is an atmosphere that enables people and the planet to thrive – stabilizing the climate with warming limited to 1.5°C and drastically reduced air pollution.	CCAC
INSPIRE: Seven strategies for ending violence against children reflects the contributions of technical experts from all core agencies, and many other partners.	2016	INSPIRE's vision is a world where all governments, with the strong participation of civil society and communities, routinely implement and monitor interventions to prevent and respond to violence against all children and adolescents and help them reach their full potential.	WHO
The global strategy for women's, children's, and adolescents' health (2016-2030)	2016	By 2030, a world in which every woman, child and adolescent in every setting realizes their rights to physical and mental health and well-being, has social and economic opportunities, and can participate fully in shaping prosperous and sustainable societies.	UN
The Global Fund Strategy 2017-2022: Investing to end epidemics	2017	A world free of the burden of AIDS, tuberculosis, and malaria with better health for all.	The Global Fund
Global Plan: Decade of Actions for Road Safety 2021-2030	2021	UN General Assembly Resolution 74/299 declared a Decade of Action for Road Safety 2021-2030, with the target to reduce road traffic deaths & injuries by at least 50% during that period	WHO/UN
Global Technical Strategy for Malaria 2016–2030	2016	The vision of WHO and the global malaria community is a world free of malaria.	WHO
Global Plan to End TB: 2018-2022	2019	We are committed to mobilizing every potential resource, every ounce of energy and every dollar necessary until we live in a world where no one dies from TB.	Stop TB Partnership
The SDG 6 Global Acceleration Framework	2020	The Framework follows a set of guiding principles such as prioritizing the vulnerable, inclusivity, conflict sensitivity, unleashing female and youth potential, planning for resilience and designing and implementing transformations based on scientific evidence.	UN Water
Plan of Action for the Global Campaign for Violence Prevention for the period 2012-2020	2012	This Plan of Action for the Global Campaign for Violence Prevention aims to unify the efforts of the main actors in international violence prevention and identify a small set of priorities for the field, by presenting six national level goals towards which efforts can be directed.	WHO
A Five-Year Action Plan on Health Employment and Inclusive Economic Growth	2017	Working for Health accelerates progress towards universal health coverage (UHC) and the 2030 Agenda by catalysing the expansion and transformation of the global health and social workforce through intersectoral actions, sustainable investments, and institutional capacity building.	WHA

Supplement 3: Guiding factors in participant decision making in round two

Domain	Based on feasibility	Based on impact on equity	Based on relevance
Research and further contextualisation of drowning prevention	28·2%	27·3%	44·5%
Best practice guidance	27·6%	25·8%	46·7%
Capacity building	30·3%	23·9%	45·8%
Engagement with health and sustainable development agendas	26·7%	31·2%	42·1%
High-level political advocacy	32·1%	30·3%	37·6%
Multisectoral action	30·0%	25·5%	44·5%
Inclusive global governance	31·2%	27·6%	41·2%
All Domains	29·4%	27·4%	43·2%
